# Supplementary material for: RNA-Based Detection of Gene Fusions in Formalin-Fixed and Paraffin-Embedded Solid Cancer Samples
Source: Cancers (Basel). 2019 Sep 5;11(9):1309. doi: 10.3390/cancers11091309 (PMC6769558; doi:10.3390/cancers11091309)
Supplement: Supplementary file 1 [file cancers-11-01309-s001.pdf]

Supplementary Materials

RNA-Based Detection of Gene Fusions in Formalin-Fixed and Paraffin-Embedded Solid Cancer Samples

Martina Kirchner, Olaf Neumann, Anna-Lena Volckmar, Fabian Stögbauer, Michael Allgäuer, Daniel Kazdal, Jan Budczies, Eugen Rempel, Regine Brandt, Suranand Babu Talla, Moritz von Winterfeld, Jonas Leichsenring, Tilmann Bochtler, Alwin Krämer, Christoph Springfeld, Peter Schirmacher, Roland Penzel, Volker Endris and Albrecht Stenzinger

Table S1. PCR Primers for gene fusions identified with either the OCAv3- or Archer-panel.

Fusion	Primer	Seq	Primer	Seq	Amplicon [bp]
AXL::CAPN15 (A19C2)	AXL F	CATGGATGAGGGTGGAGGTT	CAPN15 R	CTGGGCACACGTGAATCAC	178
BRD3::NUTM1 (B11N2)	BRD3 F	AAGAAACAGGCAGCCAAGTC	NUTM1 R	CTGGTGGGTCAGAAGTTGGT	217
ESR1-CCDC170 (E2C7)	ESR1 F	GGAGACTCGCTACTGTGCA	CCDC170 R	CCCAGACTCCTTTCCCAACT	167
ESR1-QKI (E2Q5)	ESR1 F	GGAGACTCGCTACTGTGCA	QKI R	GGCTGGTGATTTAATGTTGGC	197
ETV6::NTRK3 (E5N15)	ETV6 F	AAGCCCATCAACCTCTCTCA	NTRK3 R	GGGCTGAGGTTGTAGCACTC	206
FGFR2::INA (F17I2)	FGFR2 F	CTCCCAGAGACCAACGTTCA	INA R	GTCCTGGTATTCCCGAAAGGT	148
FNDC3B-PIK3CA (F3P2)	FNDC3B F	GCAGCTCAGCAGGTTATTCT	PIK3CA R1	GTCGTGGAGGCATTGTTCTG	177
GATM::RAF1 (G2R8)	GATM F	CTTACAACGAATGGGACCCC	RAF1 R	GTTGGGCTCAGATTGTTGGG	160
GPBP1L1::MAST2 (G6M4)	GPBP1L1 F1	CGTAGTGGAGGTGGCACA	MAST2 R1	AGGTGATGTGCTAGAGGTCA	178
HNRNPA2B1::ETV1 (H9E6)	HNRNPA 2B1 F	GGAGGATATGGTGGTGGAGG	ETV1 R	TTGATTTTCAGTGGCAGGCC	164
MYB::NFIB (M12N9)	MYB12 F	TGATGAATCTGGAATTGTTGCT G	NFIB9 R	CGTAATTTTGGACATTGGCCG	150
MYB::NFIB (M13N9)	MYB13 F	TCTTCTGCTCACACCACTGG	NFIB9 R	CGTAATTTTGGACATTGGCCG	160
SND1::BRAF (S9B9)	SND1 F	CGATTCACCTGTCCAGCATC	BRAF R	CGCTGAGGTCCTGGAGATTT	184
TBL1XR1::PIK3CA (T1P2)	TBL1XR1 F	TTTCCTTGTGCCTCCATTCC	PIK3CA R	GTCGTGGAGGCATTGTTCTG	195
TMPRSS2::ERG (T2E4)	TMPRSS2 F	CGCGGCAGGTCATATTGAA	ERG R	CCTTCCCATCGATGTTCTGG	190
WHSC1L1::FGFR1 (W1F2)	WHSC1L1 F	TGATCGCACTGACACGGC	FGFR1 R	ACAAGGCTCCACATCTCCAT	108

Table S2. Clinical and diagnostic implications of the detected gene fusions.

Fusion	Entity Where Fusion Is Detected	Reclassification Based on Molecular Analysis (AMP/OCA)	Entities Where Fusion Is Supportive of Diagnosis	Drug	Ref.	3′- Gene	5′-Gene
TMPRSS2::ERG	Prostate cancer		Prostate cancer	None		TMPRSS2 Exon 1 (NM_005656)	ERG Exon 2 (NM_182918)
TMPRSS2::ERG	Prostate cancer		Prostate cancer	None		TMPRSS2 Exon 1 (NM_005656)	ERG Exon 2 (NM_004449)
TMPRSS2::ERG	Prostate cancer		Prostate cancer	None		TMPRSS2 Exon 2 (NM_005656)	ERG Exon 4 (NM_004449)
TMPRSS2::ERG	Prostate cancer		Prostate cancer	None		TMPRSS2 Exon 2 (NM_005656)	ERG Exon 4 (NM_004449)
TMPRSS2::ERG	Prostate cancer		Prostate cancer	None		TMPRSS2 Exon 2 (NM_005656)	ERG Exon 4 (NM_004449)
TMPRSS2::ERG	Prostate cancer		Prostate cancer	None		TMPRSS2 Exon 1 (NM_005656)	ERG Exon 5 (NM_004449)
TMPRSS2::ERG	Prostate cancer		Prostate cancer	None		TMPRSS2 Exon 1 (NM_005656)	ERG Exon 4 (NM_004449)
TMPRSS2::ERG	CUP DD Prostate cancer	Prostate cancer	Prostate cancer	None		TMPRSS2 Exon 1 (NM_005656)	ERG Exon 4 (NM_004449)
TMPRSS2::ERG	Prostate cancer		Prostate cancer	None		TMPRSS2 Exon 1 (NM_005656)	ERG Exon 4 (NM_004449)
MYB::NFIB	Adenoid cystic carcinoma		Adenoid cystic carcinoma	None		MYB Exon 12 (NM_005375)	NFIB Exon 9 (NM_005596)
MYB::NFIB	CUP	Adenoid cystic carcinoma	Adenoid cystic carcinoma	None		MYB Exon 12 (NM_005375)	NFIB Exon 9 (NM_005596)
MYB::NFIB	Adenoid cystic carcinoma		Adenoid cystic carcinoma	None		MYB Exon 14 (NM_005375)	NFIB Exon 9 (NM_005596)
MYB::NFIB	Adenoid cystic carcinoma		Adenoid cystic carcinoma	None		MYB Exon 11 (NM_005375)	NFIB Exon 9 (NM_005596)
MYB::NFIB	Adenoid cystic carcinoma		Adenoid cystic carcinoma	None		MYB Exon 13 (NM_005375)	NFIB Exon 9 (NM_005596)

MYB::NFIB	Adenoid cystic carcinoma		Adenoid cystic carcinoma	None		MYB Exon 13 (NM_005375)	NFIB Exon 9 (NM_005596)
MYB::NFIB	Adenoid cystic carcinoma		Adenoid cystic carcinoma	None		MYB Exon 14 (NM_005375)	NFIB Exon 10 (NM_001282787)
DNAJB1::PRKACA	Fibrolamellular carcinoma		Fibrolamellular carcinoma	None		DNAJB1 Exon 1 (NM_006145)	PRKACA Exon 2 (NM_207518)
KIF5B::RET	CUP		Lung cancer	Cabozantinib (Pan-kinase inhibitor), Nintedanib, Sunitinib, Vandetanib (Pan-TK inhibitor)	[34–37]	KIF5B Exon 15 (NM_004521)	RET Exon 12 (NM_020630)
EML4::ALK (V2)	CUP	CUP (Biliary tract / NSCLC)	Lung cancer	e.g. Alectinib, Crizotinib (ALK inhibitor), Entrictinib (Pan-TK inhibitor)	[38–42]	EML4 Exon 20 (NM_019063)	ALK Exon 20 (NM_004304)
EML4::ALK (V3)	Lung		Lung cancer	e.g. Alectinib, Crizotinib (ALK inhibitor), Entrictinib (Pan-TK inhibitor)	[38–42]	EML4 Exon 6 (NM_019063)	ALK Exon 20 (NM_004304)
VTCN1::NRG1	Cholangiocarcinoma		None	Afatinib (ERBB2&EGFR inhibitor 2nd gen), Lapatinib (ERBB2 inhibitor)		VCTN1 Exon 2 (NM_024626)	NRG1 Exon 2 (NM_004495)
TRIM24::BRAF	CUP		None	BRAF inhibitor, MEK inhibitors (Selumetinib,Trametinib), Sorafenib	[43–45]	TRIM24 Exon 9 (NM_003852)	BRAF Exon 11 (NM_004333)
SND1::BRAF	Pancreatic carcinoma		None	BRAF inhibitor, MEK inhibitors (Selumetinib,Trametinib), Sorafenib		SND1 Exon 9 (NM_014390)	BRAF Exon 9 (NM_004333)
SND1::BRAF	Pancreatic carcinoma		None	BRAF inhibitor, MEK inhibitors (Selumetinib,Trametinib), Sorafenib		SND1 Exon 9 (NM_014390)	BRAF Exon 9 (NM_004333)
GATM::RAF1	Neuroendocrine tumor (pancreas)		None	Pan-RAF inhibitors,Sorafenib (Pan-Tk Inhibitor), U0126 (MEK Inhibitor)		GATM Exon 2 (NM_001482)	RAF1 Exon 8 (NM_002880)
ETV6::NTRK3	MASC		None	Larotrectinib, Entrectinib, IGF1R inhibitors, PI3K pathway inhibitors, Midostaurin (Pan-TK inhibitor)		ETV6 Exon 5 (NM_001987)	NTRK3 Exon 15 (NM_002530)

FGFR2::INA	Pancreatic carcinoma		None	FGFR inhibitors		FGFR2 Exon 17 (NM_000141)	INA Exon 2 (NM_032727)
WHSC1L1::FGFR1	Pancreatic carcinoma		None	None		WHSC1L1 Exon 1 (NM_0177778)	FGFR1 Exon 2 (NM_015850)
TBL1XR1::PIK3CA	Pancreatic carcinoma		None	None		TBL1XR1 Exon 1 (ENST0000045792 8)	PIK3CA Exon 2 (NM_006218)
TBL1XR1::PIK3CA	Chordoma		None	None		TBL1XR1 Exon 1 (ENST0000045792 8)	PIK3CA Exon 2 (NM_006218)
RNF130::SEPT14	CUP		None	None		RNF130 Exon 3 (NM_018434)	SEPT14 Exon 10 (NM_207366)
PTPRK::RSPO3	Pancreatic carcinoma		None	None		PTPRK Exon 1 (NM_002844)	RSPO3 Exon 2 (NM_032784)
MTMR::MAML2	Cholangiocarcinoma		None	None		MTMR Exon 2	MAML2 Exon 1
HNRNPA2B1::ET V1	Prostate cancer		Prostate cancer	None		HNRNPA2B1 Exon 9 (NM_002137)	ETV1 Exon 6 (NM_004956)
GPBP1L1::MAST2	Pancreatic cancer		None	None		GPBP1L1 Exon 6	MAST Exon 4
FNDC3B::PIK3CA	Gallbladder carcinoma		None	None		FNDC3B Exon 3 (NM_022763)	PIK3CA Exon 2 (NM_006218)
ESR1::QKI	CUP		None	None		ESR1 Exon 2 (NM_001122742)	QKI Exon 5 (NM_006775)
AXL::CAPN15	Gastric cancer		none	None		AXL Exon 19	CAPN15 Exon 2
BRD4::NUTM1	CUP	NUT Midline Tumor	NUT Midline Tumor	Birabresib	[46,47]	BRD4 Exon 14 (NM_058243)	NUTM1 Exon 2 (NM_001284293)
BRD3::NUTM1	Lung cancer (NUT Midline Tumor)	NUT Midline Tumor	NUT Midline Tumor	Birabresib	[46,47]	BRD3 Exon 11 (NM_007371)	NUTM1 Exon 2 (NM_175741)

Table S3. OCAv3 Fusion panel overview.

**A2M-ALK**	**ACBD5-RET**	**ACTG2-ALK**	**ADAMTS16-TERT**	**AFAP1-NTRK2**
A22A19	A11R12	A2A18	A8T3	A14N12
**AFAP1-RET**	**AGAP3-BRAF**	**AGAP3-BRAF**	**AGBL4-NTRK2**	**AGGF1-RAF1**
A3R12	A10B11	A9B9	A6N16	A5R8
**AGK-BRAF**	**AGTRAP-BRAF**	**AKAP13-NTRK3**	**AKAP13-RET**	**AKAP13-RET**
A2B8	A5B8	A14N14	A35R12	A36R12
**AKAP9-BRAF**	**AKAP9-BRAF**	**AKAP9-BRAF**	**AKAP9-BRAF**	**AKAP9-BRAF**
A21B10	A22B9	A28B9	A7B11	A8B9
**ALK-PTPN3**	**AP3B1-BRAF**	**AR-AR**	**AR-AR**	**AR-AR**
A11P3	A22B9	A1ins69A3	A2A3	A3A3
**AR-AR**	**AR-AR**	**AR-AR**	**AR-AR**	**AR-AR**
A3A3int4	A3A4	A3A5	A3int4A3	A4A6
**AR-AR**	**AR-AR**	**AR-AR**	**AR-AR**	**AR-AR**
A4A8	A6A8	A6A9	A8A9	E4E7
**AR-AR**	**ARHGEF2-NTRK1**	**ARMC10-BRAF**	**AR-OPHN1**	**ATAD2-ERBB4**
E7E8	A21N10	A4B11	A3O3	A8E15
**ATF7IP-JAK2**	**ATF7IP-PDGFRB**	**ATG7-BRAF**	**ATIC-ALK**	**ATP1B1-PRKACA**
A13J17	A13P11	A18B9	A7A20	A1P2
**ATP1B1-PRKACB**	**ATRNL1-ALK**	**AXL-MBIP**	**B4GALT1-RAF1**	**BAG4-FGFR1**
A1P2	A19A20	A20M4	B1R8	B1F8
**BAG4-FGFR1**	**BAIAP2L1-BRAF**	**BAIAP2L1-MET**	**BBS9-BRAF**	**BCAM-AKT2**
B2F6	B12B9	B9M15	B19B4	B13A5
**BCAN-NTRK1**	**BCL2L11-BRAF**	**BCR-FGFR1**	**BCR-JAK2**	**BCR-JAK2**
B13N11	B3B10	B4F10	B1J15	B1J17
**BCR-JAK2**	**BCR-PDGFRA**	**BCR-PDGFRA**	**BCR-PDGFRA**	**BEND5-ALK**
B1J19	B12ins12P12	B1P13	B7ins24P12	B3A20
**BICD2-JAK2**	**BIN2-PDGFRB**	**BIRC6-ALK**	**BRAF-AP3B1**	**BRAF-BRAF**
B7J13	B9P12	B10A20	B8A23	B1B11
**BRAF-BRAF**	**BRAF-BRAF**	**BRAF-BRAF**	**BRAF-CIITA**	**BRAF-MACF1**
B1B9	B3B11	B3B9	B9C6	B8M15
**BRAF-MRPS33**	**BRAF-SLC26A4**	**BRAF-SUGCT**	**BRCA1-BRCA1**	**BRCA1-BRCA1**

B1M2	B3S7	B1S13	B10B14	B10B16
**BRCA1-BRCA1**	**BRCA1-BRCA1**	**BRCA1-BRCA1**	**BRCA1-BRCA1**	**BRCA1-BRCA1**
B11B16	B13B15	B15B17	B15B18	B16B18
**BRCA1-BRCA1**	**BRCA1-BRCA1**	**BRCA1-BRCA1**	**BRCA1-BRCA1**	**BRCA1-BRCA1**
B19B21	B19B23	B20B23	B4B15	B7B12
**BRCA1-BRCA1**	**BRCA1-BRCA1**	**BRCA2-BRCA2**	**BRCA2-BRCA2**	**BRCA2-BRCA2**
B7B14	B7B9	B11B11	B11B22	B11B27
**BRCA2-BRCA2**	**BRCA2-BRCA2**	**BRCA2-BRCA2**	**BRCA2-BRCA2**	**BRD3-NUTM1**
B13B17	B1B3	B21B25	B7B10	B10N2
**BRD4-NUTM1**	**BRD4-NUTM1**	**BRD4-NUTM1**	**BTBD1-NTRK3**	**BTF3L4-BRAF**
B11N2	B14N2del585	B15N2	B4N14	B3B11
**C11orf95-RELA**	**C11orf95-RELA**	**C7orf73-BRAF**	**C8orf34-MET**	**CAD-ALK**
C3R2	C3R3	C2B9	C2M15	C35A20
**CAND1-EGFR**	**CAPRIN1-PDGFRB**	**CAPZA2-MET**	**CAPZA2-MET**	**CARS-ALK**
C4E16	C7P11	C1M6	C4M11	C17A20
**CCDC127-TERT**	**CCDC6-BRAF**	**CCDC6-PDGFRB**	**CCDC6-RET**	**CCDC6-RET**
C2T3	C1B9	C7P11	C1R11	C1R11
**CCDC6-RET**	**CCDC6-RET**	**CCDC6-RET**	**CCDC6-RET**	**CCDC6-RET**
C1R12	C1R12	C1R13	C1R9	C2R11
**CCDC6-RET**	**CCDC6-RET**	**CCDC6-RET**	**CCDC6-RET**	**CCDC6-RET**
C2R12	C5ins16R11	C8R11	C8R11	C8R12
**CCDC6-ROS1**	**CCDC88A-ALK**	**CCDC88C-PDGFRB**	**CCDC88C-PDGFRB**	**CCDC88C-PDGFRB**
C5R35	C12A20	C10P12	C12P11	C25P11
**CCDC91-BRAF**	**CCNY-BRAF**	**CD44-FGFR2**	**CD74-NRG1**	**CD74-NRG1**
C11B9	C1B10	C1F3	C6N6	C8N6
**CD74-NTRK1**	**CD74-ROS1**	**CD74-ROS1**	**CD74-ROS1**	**CD74-ROS1**
C7N10	C4R33	C6R32	C6R34	C6R35
**CDC27-BRAF**	**CDK5RAP2-PDGFRA**	**CDK6-EGFR**	**CDKN2A-CDKN2A**	**CEL-NTRK1**
C16B9	C13ins40P12	C2E23	C1C3	C7N7
**CEP85L-PDGFRB**	**CEP85L-ROS1**	**CEP89-BRAF**	**CHD9-RAD51B**	**CHTOP-NTRK1**
C11P12	C8R36	C16B9	C2R8	C5N10

**CHTOP-NTRK1**	**CIC-NUTM1**	**CLCN6-BRAF**	**CLCN6-RAF1**	**CLIP1-ALK**
C5N11	C16N4	C2B11	C2R8	C13A20
**CLIP1-ROS1**	**CLIP2-BRAF**	**CLIP4-ALK**	**CLTC-ALK**	**CLTC-ALK**
C19R36	C6B11	C12A23	C31A20	C31A20
**CLTC-ALK**	**CNTLN-RAF1**	**CNTRL-FGFR1**	**COX5A-NTRK3**	**CPSF6-FGFR1**
C31ins63A20	C5R8	C40F10	C1N15	C8int8F10
**CPSF6-PDGFRB**	**CREB3L2-PPARG**	**CTNNB1-FGFR2**	**CUL1-BRAF**	**CUX1-BRAF**
C5P11	C2P2	C1F10	C7B9	C10B9
**CUX1-FGFR1**	**CUX1-RET**	**DAB2IP-NTRK2**	**DCTN1-ALK**	**DCTN1-ALK**
C11F10	C10R12	D1N17	D26A20	D29A20
**DCTN1-MET**	**DIP2C-PDGFRA**	**DIP2C-PDGFRA**	**DNAJB1-PRKACA**	**DNAJB1-PRKACA**
D26M15	D1P10	D1P11	D1P2	D2P2
**DTD1-PDGFRB**	**DYNC1I2-BRAF**	**EBF1-JAK2**	**EBF1-PDGFRB**	**EBF1-PDGFRB**
D4P12	D7B10	E14J17	E11P11	E14P11
**EBF1-PDGFRB**	**EGFR-ACADM**	**EGFR-DYM**	**EGFR-EGFR**	**EGFR-EGFR**
E15P11	E1A3	E1D3	E13E15	E1E8
**EGFR-EGFR**	**EGFR-EGFR**	**EGFR-EGFR**	**EGFR-ERP44**	**EGFR-GNS**
E24E28	E25E18	E3E5	E1E5	E17G14
**EGFR-PDP1**	**EGFR-PSPH**	**EGFR-SEPT14**	**EGFR-VOPP1**	**EIF3E-RAD51B**
E1P2	E24P6	E24S10	E24V2	E1R5
**EIF3E-RSPO2**	**EIF3E-RSPO2**	**EIF3E-RSPO2**	**EML4-ALK**	**EML4-ALK**
E1ins351R2	E1R2	E1R3	E13A20	E13A20
**EML4-ALK**	**EML4-ALK**	**EML4-ALK**	**EML4-ALK**	**EML4-ALK**
E13ins90A20	E14A20	E14del36A20	E14ins124A20	E14ins2del52A20
**EML4-ALK**	**EML4-ALK**	**EML4-ALK**	**EML4-ALK**	**EML4-ALK**
E15A20	E17A20	E17ins30A20_V8a	E17ins65A20	E17ins68A20
**EML4-ALK**	**EML4-ALK**	**EML4-ALK**	**EML4-ALK**	**EML4-ALK**
E17int17Aint19E20	E18A20	E19A20	E20A20	E21A20
**EML4-ALK**	**EML4-ALK**	**EML4-ALK**	**EML4-ALK**	**EML4-ALK**
E2A20	E3p53insA20	E6A17	E6A18	E6A19
**EML4-ALK**	**EML4-ALK**	**EML4-ALK**	**EML4-ALK**	**EML4-BRAF**
E6aA20	E6bA20	E6ins18A20	E7A20	E6B10

**EML4-NTRK3**	**EPHB2-NTRK1**	**EPS15-BRAF**	**EPS15-MET**	**EPS15-NTRK1**
E2N14	E3N9	E22B10	E21M15	E21N9
**EPS15-NTRK1**	**ERBB2-ERBB2**	**ERBB2-GRB7**	**ERBB2-GRB7**	**ERC1-BRAF**
E21N9	E19E21	E29G10	E30G11	E12B10
**ERC1-BRAF**	**ERC1-PDGFRB**	**ERC1-PDGFRB**	**ERC1-RET**	**ERC1-RET**
E17B8	E15P10	E15P11	E11R12	E12R12
**ERC1-RET**	**ERC1-RET**	**ERC1-ROS1**	**ERLIN2-FGFR1**	**ERVK3_1-FGFR1**
E17R12	E7R12	E11R36	E8F2	E3F10
**ESR1-AKAP12**	**ESR1-AKAP12**	**ESR1-ARMT1**	**ESR1-CCDC170**	**ESR1-CCDC170**
E5A4	E6A4	E3A4	E2C10	E2C6
**ESR1-CCDC170**	**ESR1-CCDC170**	**ESR1-DAB2**	**ESR1-MTHFD1L**	**ESR1-PDE10A**
E2C7	E2C8	E6D3	E6M21	E7P6
**ESR1-POLH**	**ESR1-YAP1**	**ESRP1-RAF1**	**ETV6-FGFR3**	**ETV6-FLT3**
E6P2	E6Y4	E13R6	E5F9	E4F16
**ETV6-FLT3**	**ETV6-FLT3**	**ETV6-FLT3**	**ETV6-FLT3**	**ETV6-JAK2**
E4ins16F14del11	E5F14	E5F16	E5ins6F14	E4J16
**ETV6-JAK2**	**ETV6-JAK2**	**ETV6-JAK2**	**ETV6-JAK2**	**ETV6-NTRK3**
E4J17	E5J12	E5J17	E5J19	E4N14
**ETV6-NTRK3**	**ETV6-NTRK3**	**ETV6-NTRK3**	**ETV6-PDGFRA**	**ETV6-PDGFRB**
E4N15	E5N14	E5N15	E6P12	E4P11
**ETV6-PDGFRB**	**ETV6-PDGFRB**	**ETV6-PDGFRB**	**EZR-ERBB4**	**EZR-ROS1**
E4P9	E7ins34P12	E7P10	E12E18	E10R34
**EZR-ROS1**	**FAM114A2-BRAF**	**FAM131B-BRAF**	**FAM131B-BRAF**	**FAM131B-BRAF**
E10R35	F9B11	F1B10	F2B9	F3B9
**FAT1-NTRK3**	**FCHSD1-BRAF**	**FGFR1-ADAM32**	**FGFR1-NTM**	**FGFR1OP2-FGFR1**
F2N7	F13B9	F17A14	F1N2	F4F10
**FGFR1OP-FGFR1**	**FGFR1OP-FGFR1**	**FGFR1OP-FGFR1**	**FGFR1-PLAG1**	**FGFR1-PLAG1**
F5F10	F6F10	F7F10	F1P2	F1P3
**FGFR1-PLAG1**	**FGFR1-PLAG1**	**FGFR1-TACC1**	**FGFR1-TACC1**	**FGFR1-ZNF703**
F2P2	F2P3	F17T7	F18T7	F14Z2
**FGFR2-AFF3**	**FGFR2-AHCYL1**	**FGFR2-BICC1**	**FGFR2-BICC1**	**FGFR2-BICC1**

F17A8	F17A2	F17B18	F17B2	F17B3
**FGFR2-CASP7**	**FGFR2-CCAR2**	**FGFR2-CCDC6**	**FGFR2-CCDC6**	**FGFR2-CIT**
F17C2	F17C4	F17C1	F17C2	F17C23
**FGFR2-COL14A1**	**FGFR2-CREB5**	**FGFR2-FAM76A**	**FGFR2-KCTD1**	**FGFR2-MGEA5**
F17C34	F17C8	F17F2	F17K2	F17M12
**FGFR2-NOL4**	**FGFR2-OFD1**	**FGFR2-PPHLN1**	**FGFR2-SHTN1**	**FGFR2-TACC3**
F17N7	F17O3	F17P3	F17S7	F17T11
**FGFR2-TXLNA**	**FGFR2-USP10**	**FGFR3-AES**	**FGFR3-BAIAP2L1**	**FGFR3-ELAVL3**
F17T6	F17del11U5	F17A2	F17B2	F17E2
**FGFR3-FBXO28**	**FGFR3-JAKMIP1**	**FGFR3-TACC3**	**FGFR3-TACC3**	**FGFR3-TACC3**
F17F4	F17J4	F14T11	F15T11	F16T10
**FGFR3-TACC3**	**FGFR3-TACC3**	**FGFR3-TACC3**	**FGFR3-TACC3**	**FGFR3-TACC3**
F16T11	F17ins1T10	F17intron17T4	F17T10	F17T11
**FGFR3-TACC3**	**FGFR3-TACC3**	**FGFR3-TACC3**	**FGFR3-TACC3**	**FGFR3-TACC3**
F17T13	F17T14	F17T4	F17T5	F17T6
**FGFR3-TACC3**	**FGFR3-TACC3**	**FGFR3-TACC3**	**FGFR3-TACC3**	**FGFR3-TACC3**
F17T7	F17T8	F17T9	F18T1	F18T10
**FGFR3-TACC3**	**FGFR3-TACC3**	**FGFR3-TACC3**	**FGFR3-TACC3**	**FGFR3-TACC3**
F18T11	F18T11del5	F18T4and5	F18T7	TruncatedF17T4
**FIP1L1-PDGFRA**	**FIP1L1-PDGFRA**	**FIP1L1-PDGFRA**	**FIP1L1-PDGFRA**	**FIP1L1-PDGFRA**
F10int10P12del106	F10int10P12del22	F10int10P12del67	F10P12del47	F11P12del24
**FIP1L1-PDGFRA**	**FIP1L1-PDGFRA**	**FIP1L1-PDGFRA**	**FIP1L1-PDGFRA**	**FIP1L1-PDGFRA**
F11P12del26	F11P12del45	F11P12del77	F11P12del77	F12P12del107
**FIP1L1-PDGFRA**	**FIP1L1-PDGFRA**	**FIP1L1-PDGFRA**	**FIP1L1-PDGFRA**	**FIP1L1-PDGFRA**
F12P12del84	F13ins11P12del99	F13insP12del91	F13P12del71	F13P12del75
**FIP1L1-PDGFRA**	**FIP1L1-PDGFRA**	**FKBP15-RET**	**FN1-ALK**	**FN1-ALK**
F16ins16P12del71	F9P12del38	F25R12	F20A19	F23A19
**FN1-FGFR1**	**FN1-FGFR1**	**FN1-FGFR1**	**FN1-FGFR1**	**FN1-FGFR1**
F22F3	F22F4	F23F3	F23F4	F28F5
**FNDC3B-PIK3CA**	**FOXP1-PDGFRA**	**FXR1-BRAF**	**FYCO1-RAF1**	**GATM-BRAF**
F3P2	F16P12	F13B10	F11R6	G2B11
**GFPT1-ALK**	**GHR-BRAF**	**GIT2-PDGFRB**	**GLIS3-TERT**	**GNAI1-BRAF**

G18A20	G1B10	G12P11	G3T3	G1B10
**GOLGA4-PDGFRB**	**GOLGA4-RAF1**	**GOLGA5-RET**	**GOLGB1-PDGFRB**	**GOPC-ROS1**
G10P11	G16R8	G7R12	G10P12	G4R36
**GOPC-ROS1**	**GRHL2-RSPO2**	**GTF2I-BRAF**	**GTF2IRD1-ALK**	**GTF3C2-ALK**
G8R35	G8R2	G4B10	G7A20	G1A18
**HACL1-RAF1**	**HERPUD1-BRAF**	**HIP1-ALK**	**HIP1-ALK**	**HIP1-ALK**
H16R8	H4B7	H21A20	H28A20	H30A20
**HIP1-PDGFRB**	**HLA_A-ROS1**	**HMBS**	**HMGA2-RAD51B**	**HMGA2-RAD51B**
H30P11	H7R34	ENCTRL	H3R11	H3R8
**HOOK3-RET**	**IRF2BP2-NTRK1**	**ITGB7**	**KANK1-PDGFRB**	**KANK2-ALK**
H11R12	I1N10	ENCTRL	K2P9	K4A16
**KCNQ5-ALK**	**KCTD7-BRAF**	**KCTD7-BRAF**	**KDELR2-ROS1**	**KDELR2-ROS1**
K1A10	K3B8	K4B8	K5R35	K5Rintron34
**KDM7A-BRAF**	**KDR-PDGFRA**	**KIAA1468-RET**	**KIAA1549-BRAF**	**KIAA1549-BRAF**
K11B11	K13ins35P10	K10R12	K12B11	K12B9
**KIAA1549-BRAF**	**KIAA1549-BRAF**	**KIAA1549-BRAF**	**KIAA1549-BRAF**	**KIAA1549-BRAF**
K13B9	K14B11	K14B9	K15B10	K15B11
**KIAA1549-BRAF**	**KIAA1549-BRAF**	**KIAA1549-BRAF**	**KIAA1549-BRAF**	**KIAA1549-BRAF**
K15B9	K16B10	K17B10	K18B9	K9B9
**KIAA1598-ROS1**	**KIF5B-ALK**	**KIF5B-ALK**	**KIF5B-ALK**	**KIF5B-ALK**
K11R36	K15A20	K15A20	K17A20	K24A19
**KIF5B-ALK**	**KIF5B-PDGFRA**	**KIF5B-RET**	**KIF5B-RET**	**KIF5B-RET**
K24A20	K23P12	K15R11	K15R12	K16R12
**KIF5B-RET**	**KIF5B-RET**	**KIF5B-RET**	**KIF5B-RET**	**KIF5B-RET**
K18R12	K22R12	K23R12	K24R11	K24R8
**KLC1-ALK**	**KLHL7-BRAF**	**KTN1-ALK**	**KTN1-RET**	**LMNA-NTRK1**
K9A20	K5B9	K43A19	K29R12	L10N11
**LMNA-NTRK1**	**LMNA-NTRK1**	**LMNA-NTRK1**	**LMNA-NTRK1**	**LMNA-NTRK1**
L10N12	L11N11	L2N11	L3N11	L5N10
**LMNA-NTRK1**	**LMNA-RAF1**	**LRIG3-ROS1**	**LRP1**	**LRRFIP1-FGFR1**
L6N12	L10R8	L16R35	ENCTRL	L8F10
**LRRFIP1-MET**	**LSM12-BRAF**	**LSM14A-BRAF**	**LYN-NTRK3**	**MACF1-BRAF**

L19M15	L3B9	L9B9	L8N14	M60B9
**MAD1L1-BRAF**	**MAD1L1-BRAF**	**MCFD2-ALK**	**MDM4-MDM4**	**MDM4-MDM4**
M16B9	M17B10	M1A20	M2M11	M3M10
**MDM4-MDM4**	**MDM4-MDM4**	**MDM4-MDM4**	**MDM4-MDM4**	**MEMO1-ALK**
M5M10	M5M7	M7M10	M8M10	M2A7
**MET-MET**	**MET-MET**	**MET-MET**	**MIR143HG-NOTCH1**	**MKRN1-BRAF**
M13M15	M17M20	M18M20	M1N27	M4B11
**MKRN1-BRAF**	**MPRIP-NTRK1**	**MPRIP-NTRK1**	**MPRIP-NTRK1**	**MPRIP-PDGFRB**
M4B9	M14N12	M18N12	M21N12	M20P12
**MPRIP-RAF1**	**MRPL13**	**MRPL24-NTRK1**	**MRPL24-NTRK1**	**MSN-ALK**
M22R8	ENCTRL	M1N9	M1N9	M11A20
**MSN-ALK**	**MSN-ROS1**	**MTMR12-TERT**	**MYB-ESR1**	**MYBL1-NFIB**
M11int12A20	M9R34	M7T3	M15E8	M14N11
**MYBL1-NFIB**	**MYBL1-NFIB**	**MYBL1-NFIB**	**MYBL1-NFIB**	**MYBL1-YTHDF3**
M15N11	M8N11	M8N12	M9N11	M8Y4int3
**MYBL1-YTHDF3**	**MYB-NFIB**	**MYB-NFIB**	**MYB-NFIB**	**MYB-NFIB**
M9Y4int3	M13N11	M13N12	M15N10	M15N11
**MYB-NFIB**	**MYB-NFIB**	**MYB-NFIB**	**MYB-NFIB**	**MYB-NFIB**
M15N12	M15N8	M15N9	M8N11	M8N12
**MYB-NFIB**	**MYB-PCDHGA1**	**MYB-QKI**	**MYB-QKI**	**MYB-QKI**
M8N9	M9P2	M11Q5	M15Q5	M9Q5
**MYB-QKI**	**MYB-TYK2**	**MYC**	**MYH13-RET**	**MYH9-ALK**
M9Q6	M6T18	ENCTRL	M35R12	M34A20del23
**MYH9-ALK**	**MYO18A-FGFR1**	**MYO18A-PDGFRB**	**MYO5A-ROS1**	**MYRIP-BRAF**
M9A6ins10	M33F10	M41P10	M23R35	M16B9
**MZT1-BRAF**	**NACC2-NTRK2**	**NAV1-NTRK2**	**NCOA1-ALK**	**NCOA4-RET**
M2B11	N4N13	N15N11	N21A1	N6R12
**NCOA4-RET**	**NCOR2-ROS1**	**NDE1-PDGFRB**	**NF1-ABCB5**	**NF1-ACACA**
N7R12	N7R36	N6P11	N48A5	N1A42
**NF1-ASIC2**	**NF1-ATAD5**	**NF1-PSMD11**	**NFASC-NTRK1**	**NFKB2-ROS1**
N27A2	N5A11	N5P2	N20N10	N13R36

**NIN-PDGFRB**	**NOTCH1-GABBR2**	**NOTCH1-NUP214**	**NOTCH1-SDCCAG3**	**NOTCH1-SNHG7**
N30P12	N30G14	N2N25	N21S5	N2S4
**NPC2-RAD51B**	**NPM1-ALK**	**NSD1-NOTCH4**	**NTRK1-DYNC2H1**	**NTRK1-NTRK1**
N1R9	N4A20	N14N18	N17D85	N6N8
**NTRK3-HOMER1**	**NUB1-BRAF**	**NUDCD3-BRAF**	**NUP214-BRAF**	**OFD1-JAK2**
N17H2	N3B9	N4B9	N21B10	O21J13
**OXR1-MET**	**PAPD7-RAF1**	**PAPSS1-BRAF**	**PARK2-FGFR2**	**PAX5-JAK2**
O9M13	P11R10	P5B9	P9F11	P5J19
**PAX8-PPARG**	**PAX8-PPARG**	**PAX8-PPARG**	**PAX8-PPARG**	**PCM1-JAK2**
P10P2	P7P2	P8P2	P9P2	P23J12
**PCM1-JAK2**	**PCM1-JAK2**	**PCM1-JAK2**	**PCM1-JAK2**	**PCM1-JAK2**
P24J17	P26J9	P28J11	P29J13	P35J11
**PCM1-JAK2**	**PCM1-JAK2**	**PCM1-JAK2**	**PCM1-JAK2**	**PCM1-NRG1**
P36J11	P36J11ins12	P36J9	P36J9ins3	P2N8
**PCM1-RET**	**PCNX-RAD51B**	**PDE4DIP-PDGFRB**	**PDE7A-NRG1**	**PDHX-FGFR2**
P29R12	P1R8	P16P11	P3N6	P1F7
**PDZRN3-RAF1**	**PLIN3-BRAF**	**PPFIBP1-ALK**	**PPFIBP1-ALK**	**PPFIBP1-JAK2**
P5R8	P1B9	P12A20	P8A20ins49	P12J19
**PPFIBP1-MET**	**PPFIBP1-ROS1**	**PPL-NTRK1**	**PPL-NTRK1**	**PPL-NTRK1**
P9M15	P9R35	P12N13	P22N10	P22N11
**PPM1G-ALK**	**PPP4R3B-ALK**	**PRKAR1A-ALK**	**PRKAR1A-RET**	**PRKG2-PDGFRB**
P1A18	P9A2	P2A20	P7R12	P3P12
**PRKG2-PDGFRB**	**PTEN-BTAF1**	**PTEN-SHROOM4**	**PTEN-SHROOM4**	**PTPRK-RSPO3**
P6P12	P2B2	P2S3	P3S4	P1R2
**PTPRK-RSPO3**	**PTPRZ1-MET**	**PTPRZ1-MET**	**PTPRZ1-MET**	**PWWP2A-ROS1**
P7R2	P1M2	P3M2	P8M2	P1R36
**QKI-NTRK2**	**QKI-RAF1**	**RABEP1-PDGFRB**	**RABGAP1L-NTRK1**	**RAD18-BRAF**
Q6N16	Q3R8	R14P11	R14N16	R7B10
**RAF1-C9orf153**	**RANBP2-ALK**	**RANBP2-FGFR1**	**RB1-RB1**	**RB1-RB1**
R14C2	R18A20	R20F10int9	R20R24	R21R23
**RB1-RB1**	**RBMS3-BRAF**	**RBPMS-NTRK3**	**RNF11-BRAF**	**RNF130-BRAF**

R21R25	R11B11	R5N14	R1B11	R3B9
**RNF213-ALK**	**RNF213-NTRK1**	**RP2-BRAF**	**RUFY2-RET**	**SART3-PDGFRB**
R20A20	R15N12	R3B10	R9R12	S15P11
**SCAF11-PDGFRA**	**SDC4-NRG1**	**SDC4-ROS1**	**SDC4-ROS1**	**SDC4-ROS1**
S1P2	S4N6	S2R32	S2R34	S4R32
**SDC4-ROS1**	**SEC16A-NOTCH1**	**SEC16A-NOTCH1**	**SEC31A-ALK**	**SEC31A-ALK**
S4R34	S1N27	S1N28	S21A20	S22A20
**SEC31A-JAK2**	**SEC61G-EGFR**	**SLC12A7-BRAF**	**SLC12A7-TERT**	**SLC34A2-ROS1**
S22J17	S2E9	S17B11	S1T3	S13R32
**SLC34A2-ROS1**	**SLC34A2-ROS1**	**SLC34A2-ROS1**	**SLC34A2-ROS1**	**SLC3A2-NRG1**
S13R34	S13R36	S4R32	S4R34	S5N6
**SLC45A3-BRAF**	**SLC45A3-ERG**	**SLC45A3-FGFR2**	**SLC45A3-FGFR2**	**SLMAP-NTRK2**
S1B8	S1E4	S1F1	S1F2	S14N16
**SND1-BRAF**	**SND1-BRAF**	**SND1-BRAF**	**SND1-BRAF**	**SND1-BRAF**
S10B11	S10B9	S11B11	S14B11	S14B9
**SND1-BRAF**	**SND1-BRAF**	**SND1-BRAF**	**SND1-BRAF**	**SNX19-FGFR2**
S16B9	S18B10	S9B2	S9B9	S7F7
**SNX19-FGFR2**	**SOX6-BRAF**	**SOX6-BRAF**	**SPAG9-JAK2**	**SPECC1L-RET**
S8F7	S5B9	S6B9	S25J19	S10R11
**SPECC1L-RET**	**SPECC1-PDGFRB**	**SPTBN1-FLT3**	**SQSTM1-ALK**	**SQSTM1-FGFR1**
S10R12	S3P11	S3F14	S5A20	S6F10
**SQSTM1-NTRK1**	**SQSTM1-NTRK1**	**SQSTM1-NTRK2**	**SRGAP3-RAF1**	**SRGAP3-RAF1**
S2N10	S5N10	S5N17	S11R8	S12R10
**SSBP2-JAK2**	**SSBP2-JAK2**	**SSBP2-JAK2**	**SSBP2-JAK2**	**SSBP2-NTRK1**
S10J18	S4J11	S5J11	S8J18	S12N12
**STK32B-ALK**	**STRN3-BRAF**	**STRN3-JAK2**	**STRN-ALK**	**STRN-PDGFRA**
S11A20	S3B10	S9J17	S3A20	S6P12
**STRN-PDGFRA**	**TANK-BRAF**	**TAX1BP1-BRAF**	**TBL1XR1-PIK3CA**	**TBL1XR1-RET**
S6P12	T4B9	T8B11	T1P2	T9R11
**TBL1XR1-RET**	**TBP**	**TENM4-NRG1**	**TERF2-JAK2**	**TERT-ALK**
T9R12	ENCTRL	T12N2	T8J19	T11A5
**TFG-ALK**	**TFG-ALK**	**TFG-ALK**	**TFG-ALK**	**TFG-MET**

T4A20	T5A20	T6A20	T7A19	T5M15
**TFG-NTRK1**	**TFG-NTRK1**	**TFG-ROS1**	**TMEM106B-ROS1**	**TMEM178B-BRAF**
T6N10	T6N14	T4R35	T3R35	T2B9
**TMPRSS2-BRAF**	**TMPRSS2-ERG**	**TMPRSS2-ERG**	**TMPRSS2-ERG**	**TMPRSS2-ERG**
T3B11	T1E2	T1E3	T1E4	T1E5
**TMPRSS2-ERG**	**TMPRSS2-ERG**	**TMPRSS2-ERG**	**TMPRSS2-ERG**	**TMPRSS2-ERG**
T1E6	T1EIIIc_4	T2E2	T2E4	T2E5
**TMPRSS2-ERG**	**TMPRSS2-ERG**	**TMPRSS2-ERG**	**TMPRSS2-ERG**	**TMPRSS2-ERG**
T2EIIIc_4	T3E4	T4E4	T4E5	T5E4
**TMPRSS2-ERG**	**TMPRSS2-ETV1**	**TMPRSS2-ETV1**	**TMPRSS2-ETV1**	**TMPRSS2-ETV1**
T5E5	T1bE4	T1E4	T1E5	T2E5
**TMPRSS2-ETV4**	**TMPRSS2-ETV5**	**TMPRSS2-ETV5**	**TMPRSS2-ETV5**	**TNIP1-PDGFRB**
T1bE3	T1bE2	T1E2	T3E2	T14P11
**TNKS2-PDGFRA**	**TP53BP1-PDGFRB**	**TP53-NTRK1**	**TP53-NTRK1**	**TP53-NTRK1**
T25P12	T23P11	T10N9	T11N9	T8N9
**TP53-NTRK1**	**TPM1-ALK**	**TPM3-ALK**	**TPM3-JAK2**	**TPM3-NTRK1**
T9N9	T8A20	T7A20	T7J17	T7N10
**TPM3-NTRK1**	**TPM3-NTRK1**	**TPM3-NTRK1**	**TPM3-NTRK1**	**TPM3-PDGFRB**
T7N12	T7N13	T7N7	T8N10	T7P11
**TPM3-ROS1**	**TPM3-ROS1**	**TPM4-ALK**	**TPR-ALK**	**TPR-ALK**
T3R36	T7R35	T7A20	T15A20	T4A20
**TPR-FGFR1**	**TPR-JAK2**	**TPR-MET**	**TPR-NTRK1**	**TPR-NTRK1**
T22F10	T39J17	T4M15	T16int9N10	T21N10
**TPR-NTRK1**	**TPR-NTRK1**	**TPR-NTRK1**	**TRAF1-ALK**	**TRAK1-RAF1**
T21N9	T6N12	T6N12	T6A20	T9R8
**TRIM24-BRAF**	**TRIM24-BRAF**	**TRIM24-BRAF**	**TRIM24-BRAF**	**TRIM24-BRAF**
T10B9	T11B2	T3B10	T3B11	T5B8
**TRIM24-BRAF**	**TRIM24-FGFR1**	**TRIM24-NTRK2**	**TRIM24-NTRK2**	**TRIM24-RET**
T9B9	T11F10	T12N15	T12N16	T9R12
**TRIM27-RET**	**TRIM33-RAF1**	**TRIM33-RET**	**TRIM33-RET**	**TRIM33-RET**
T3R12	T9R10	T11R12	T15R12	T16R12
**TRIM4-BRAF**	**TRIM4-MET**	**TRIO-TERT**	**TRIP11-PDGFRB**	**TRMT61B-ALK**

T6B10	T6M15	T33T2	T16P11	T1A9
**TSEN2-PPARG**	**TSEN2-PPARG**	**TTLL7-TERT**	**UBE2L3-KRAS**	**UBN2-BRAF**
T5P4	T6P6	T1T3	U3K2	U3B11
**VAMP2-NRG1**	**VCL-ALK**	**VCL-NTRK2**	**WASF2-FGR**	**WDR48-PDGFRB**
V4N4	V16A20	V16N12	W1F2	W9P12
**WHSC1L1-FGFR1**	**WHSC1L1-FGFR1**	**WHSC1L1-NUTM1**	**WIPF2-ERBB2**	**YWHAE-ROS1**
W14F5	W1F2	W7N2	W1E4	Y4R36
**ZC3HAV1-BRAF**	**ZC3HAV1-BRAF**	**ZCCHC8-ROS1**	**ZEB2-PDGFRB**	**ZKSCAN1-MET**
Z3B10	Z7B11	Z2R36	Z9P9	Z3M15
**ZKSCAN5-BRAF**	**ZMYM2-FGFR1**	**ZMYND8-RELA**	**ZNF226-AKT2**	**ZSCAN30-BRAF**
Z2B9	Z17F10	Z21R2	Z2A5	Z3B10

Genes are given in **bold**, exon break points are listed below.

Table S4. Genes included with anchor primers in the Archer FusionPlex Solid Tumor panel.

**Assay Targets Solid Tumor Panel**			
AKT3	EWSR1	NOTCH1/2	RAF1
ALK	FGFR1/2/3	NRG1	RELA
ARHGAP26	FGR	NTRK1/2/3	RET
AXL	INSR	NUMBL	ROS1
BRAF	MAML2	NUTM1	RSPO2/3
BRD3/4	MAST1/2	PDGFRA/B	TERT
EGFR	MET	PIK3CA	TFE3
ERG	MSMB	PKN1	TFEB
ESR1	MUSK	PPARG	THADA
ETV1/4/5/6	MYB	PRKCA/B	TMPRSS2

References

34. Gautschi, O.; Milia, J.; Filleron, T.; Wolf, J.; Carbone, D.P.; Owen, D.; Camidge, R.; Narayanan, V.; Doebele, R.C.; Besse, B.; et al. Targeting RET in Patients With RET-Rearranged Lung Cancers: Results From the Global, Multicenter RET Registry. *J. Clin. Oncol.* **2017**, *35*, 1403–1410.
35. Takeda, M.; Sakai, K.; Okamoto, K.; Hayashi, H.; Tanaka, K.; Shimizu, T.; Nishio, K.; Nakagawa, K. Genome sequencing for nonsmall-cell lung cancer identifies a basis for nintedanib sensitivity. *Ann. Oncol.* **2016**, *27*, 748–750.
36. Kohno, T.; Ichikawa, H.; Totoki, Y.; Yasuda, K.; Hiramoto, M.; Nammo, T.; Sakamoto, H.; Tsuta, K.; Furuta, K.; Shimada, Y.; et al. KIF5B-RET fusions in lung adenocarcinoma. *Nat. Med.* **2012**, *18*, 375–377.

37. Lipson, D.; Capelletti, M.; Yelensky, R.; Otto, G.; Parker, A.; Jarosz, M.; Curran, J.A.; Balasubramanian, S.; Bloom, T.; Brennan, K.W.; et al. Identification of new ALK and RET gene fusions from colorectal and lung cancer biopsies. *Nat. Med.* **2012**, *18*, 382–384.
38. Lovly, C.M.; McDonald, N.T.; Chen, H.; Ortiz-Cuaran, S.; Heukamp, L.C.; Yan, Y.; Florin, A.; Ozretic, L.; Lim, D.; Wang, L.; et al. Rationale for co-targeting IGF-1R and ALK in ALK fusion-positive lung cancer. *Nat. Med*. **2014**, *20*, 1027–1034.
39. Hrustanovic, G.; Olivas, V.; Pazarentzos, E.; Tulpule, A.; Asthana, S.; Blakely, C.M.; Okimoto, R.A.; Lin, L.; Neel, D.S.; Sabnis, A.; et al., RAS-MAPK dependence underlies a rational polytherapy strategy in EML4-ALK-positive lung cancer. *Nat. Med.* **2015**, *21*, 1038–1047.
40. Crystal, A.S.; Shaw, A.T.; Sequist, L.V.; Friboulet, L.; Niederst, M.J.; Lockerman, E.L.; Frias, R.L.; Gainor, J.F.; Amzallag, A.; Greninger, P.; et al. Patient-derived models of acquired resistance can identify effective drug combinations for cancer. *Science* **2014**, *346*, 1480–1486.
41. Socinski, M.A.; Goldman, J.; El-Hariry, I.; Koczywas, M.; Vukovic, V.; Horn, L.; Paschold, E.; Salgia, R.; West, H.; Sequist, L.V.; et al. A multicenter phase II study of ganetespib monotherapy in patients with genotypically defined advanced non-small cell lung cancer. *Clin. Cancer Res.* **2013**, *19*, 3068–3077.
42. Seto, T.; Kiura, K.; Nishio, M.; Nakagawa, K.; Maemondo, M.; Inoue, A.; Hida, T.; Yamamoto, N.; Yoshioka, H.; Harada, M.; et al. CH5424802 (RO5424802) for patients with ALK-rearranged advanced non-small-cell lung cancer (AF-001JP study): a single-arm, open-label, phase 1–2 study. *Lancet Oncol.* **2013**, *14*, 590–598.
43. Nakaoku, T.; Tsuta, K.; Ichikawa, H.; Shiraishi, K.; Sakamoto, H.; Enari, M.; Furuta, K.; Shimada, Y.; Ogiwara, H.; Watanabe, S.; et al. Druggable oncogene fusions in invasive mucinous lung adenocarcinoma. *Clin. Cancer Res.* **2014**, *20*, 3087–3093.
44. Hutchinson, K.E.; Lipson, D.; Stephens, P.J.; Otto, G.; Lehmann, B.D.; Lyle, P.L.; Vnencak-Jones, C.L.; Ross, J.S.; Pietenpol, J.A.; Sosman, J.A.; et al. BRAF fusions define a distinct molecular subset of melanomas with potential sensitivity to MEK inhibition. *Clin. Cancer Res.* **2013**, *19*, 6696–6702.
45. Palanisamy, N.; Ateeq, B.; Kalyana-Sundaram, S.; Pflueger, D.; Ramnarayanan, K.; Shankar, S.; Han, B.; Cao, Q.; Cao, X.; Suleman, K.; et al. Rearrangements of the RAF kinase pathway in prostate cancer, gastric cancer and melanoma. *Nat. Med.* **2010**, *16*, 793–798.
46. Lewin, J.; Soria, J.C.; Stathis, A.; Delord, J.P.; Peters, S.; Awada, A.; Aftimos, P.G.; Bekradda, M.; Rezai, K.; Zeng, Z.; et al. Phase Ib Trial With Birabresib, a Small-Molecule Inhibitor of Bromodomain and Extraterminal Proteins, in Patients With Selected Advanced Solid Tumors. *J. Clin Oncol.* **2018**, *36*, 3007–3014.
47. Xu, Y.; Vakoc, C.R. Targeting Cancer Cells with BET Bromodomain Inhibitors. *Cold Spring Harb. Perspect. Med.* **2017**, *7*, a026674.

© 2019 by the authors. Licensee MDPI, Basel, Switzerland. This article is an open access article distributed under the terms and conditions of the Creative Commons Attribution (CC BY) license (http://creativecommons.org/licenses/by/4.0/).
